# Supplementary material for: Photochromic Fluorophores Enable Imaging of Lowly Expressed Proteins in the Autofluorescent Fungus Candida albicans
Source: mSphere. 2021 Mar 17;6(2):10.1128/msphere.00146-21. doi: 10.1128/msphere.00146-21 (PMC8546692; doi:10.1128/msphere.00146-21)
Supplement: TABLE S1 [file msphere.00146-21-st001.pdf]

Name	Sequence (5'->3')
FW_CeffDronpa_pFA6	ctgaagcttcgtacgctgcagATGTCAGTTATTAAACCAGATATG
RV_CeffDronpa_pFA6	catgcatttacttataatggcgcgccTTATTTAGCTTGTCTTGGTAATTC
FW_GCN5_CeffDronpa	GCTTATAATTCGGAAACCACAACATATTATAAAAACGCAAATAAACTAGAAA<br>AGTTTATGAATAATAAATTGAAAGACTGTAGTTTTGTAggtgctggcgcaggtgctgg<br>tgctggcgcaggtgctATGTCAGTTATTAAACCAGAT
RV_SAT1_pFA6_GCN5	ATAACCTCCCCACAATTCTCAAAGCAACCAAATTTCAGTACTATATGGTATTT<br>CGGCCAAGAATCACATATCATATATTCGTCAAAACAGttaggcgtcatcctgtgctc
FW_ERG11_CeffDronpa	GATGGTTATAAAGTGCCTGACCCTGATTATAGTTCAATGGTGGTTTTACCTA<br>CTGAACCAGCAGAAATCATTTGGGAAAAAGAGAAACTTGTATGTTTggtgctg<br>gcgcaggtgctggtgctggcgcaggtgctATGTCAGTTATTAAACCAGATATG
RV_SAT1_pFA6_ERG11	GTATGTATATGTATATGTGTATATGTGTATATATGTTAATCCAACTAAGTAAC<br>AAAATGAAAACAATCTGAACACTGAATCGAAAGAAAGTTGCCGTTTTATTAG<br>GCGTCATCCTGTGCTC
